# Supplementary material for: From Kohn–Sham to Many-Electron Energies via Step Structures in the Exchange-Correlation Potential
Source: J Chem Theory Comput. 2021 Feb 17;17(3):1390–407. doi: 10.1021/acs.jctc.0c01093 (PMC8363072; doi:10.1021/acs.jctc.0c01093)
Supplement: Supplementary file 1 — ct0c01093_si_001.pdf [file ct0c01093_si_001.pdf]

Supplemental Material for the article: "From Kohn-Sham to many-electron energies via step structures in the exchange-correlation potential"

Eli Kraisler,[1,*] M. J. P. Hodgson,[2,3,†] and E. K. U. Gross[1]

[1] *Fritz Haber Center for Molecular Dynamics and Institute of Chemistry, The Hebrew University of Jerusalem, 9091401 Jerusalem, Israel*

[2] *Department of Physics, Durham University, South Road, Durham, DH1 3LE, United Kingdom*

[3] *Max-Planck-Institut für Mikrostrukturphysik, Weinberg 2, D-06120 Halle, Germany*

(Dated: September 3, 2020)

In this document we provide more details as to the inversion procedure of densities obtained for atoms and ions within common exchange-correlation (xc) approximations. The key results are presented in the main text, Section VII. The data provided here complements the main results, giving more technical details and peripheral information.

For the Li ion with $N = N_0 + \alpha$ electrons, where $N_0 = 2$ and $\alpha \in [0, 1]$, one obtains the Kohn-Sham (KS) potentials depicted in Fig. 1, by means of numerical inversion. The figure shows raw data, prior to any alignment procedure, as detailed below. The inversion is performed on the ensemble density $n(\mathbf{r}; N) = (1-\alpha)n(\mathbf{r}; N_0)+\alpha n(\mathbf{r}; N_0+1)$, where the integer-number densities $n(\mathbf{r}; N_0)$ and $n(\mathbf{r}; N_0 + 1)$ were obtained within the local density approximation (LDA). As mentioned in the main text, conversion of the inversion procedure was required for $r < 30$ Bohr, hence the numerical artefact that is observed for higher values of r.

As it always happens in cases of numerical inversion, the resultant potentials are obtained up to a constant.

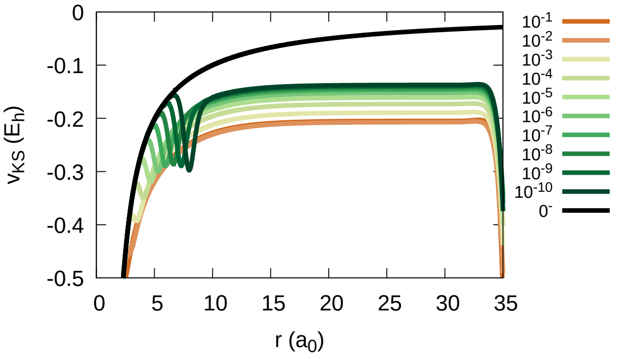

FIG. 1. Kohn-Sham potentials (raw data) for the Li ion with $2+\alpha$ electrons (see Legend), obtained by numerical inversion, relying on local density approximation (LDA) integer-electron densities.

The first choice of such a constant (for each value of α) presented in the main text is such that the KS potential approaches 0 at infinity. The way this requirement is enforced here is *not* by increasing L (the high limit of the variable r) to very high values, but rather by analysing the asymptotic behaviour of the resultant KS potentials. Fortunately, in our case the asymptotic behaviour is rather clear.

Figure 2 shows the Hartree-exchange-correlation (Hxc) potentials (obtained by subtracting the external potential, $-Z/r$, from all the potentials of Fig. 1), at far distances, as a function of $1/r$. It is easy to see that in the converged region, $r < 30$ Bohr, i.e., $1/r > 0.033\,\text{Bohr}^{-1}$, the potential $v_{\text{Hxc}}(r) \approx a \cdot \frac{1}{r} + b$, with a and b being different for each α. The values of a and b were found by linear fitting of the potentials to a straight line (in terms of $1/r$), at two points: $r = 20$ and 30 Bohrs (equivaent of $1/r = 0.033$ and 0.05 Bohr^{-1}), denoted in Fig. 2 by two vertical lines. These values of a and b are given in Table I.

The fact that the Hxc potentials obtained by inversion (invLDA) decay with a power law, and not expoenetially, as is usually expected in (semi-)local functionals, such as the LDA, is a significant improvement, and is attributed

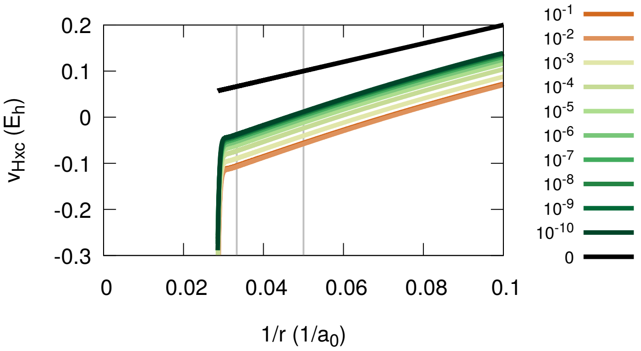

FIG. 2. Hartree-exchange-correlation (Hxc) potentials for the Li ion with $2 + \alpha$ electrons (see Legend), as a function of $1/r$. The potentials are obtained by numerical inversion, relying on local density approximation (LDA) integer-electron densities. Distances corresponding to $r = 20$ and 30 Bohrs ($1/r = 0.05$ and 0.033) are marked by vertical lines.

* These authors contributed equally; Author to whom correspondence should be addressed:eli.kraisler@mail.huji.ac.il

† These authors contributed equally

	$v_{\rm H}(r)$		$v_{\rm xc}(r)$	
α	a	b	a	b
0	2.0000000000	0	0.00000	0.00000
1e-10	2.0000000001	0	0.95239	-0.13568
1e-09	2.0000000010	0	0.95225	-0.13753
1e-08	2.0000000100	0	0.95178	-0.14033
1e-07	2.0000001000	0	0.95100	-0.14449
1e-06	2.0000010000	0	0.95108	-0.15061
1e-05	2.0000100000	0	0.95120	-0.15934
1e-04	2.0001000000	0	0.95126	-0.17153
1e-03	2.0010000000	0	0.95059	-0.18777
1e-02	2.0100000000	0	0.94227	-0.20573
1e-01	2.1000000000	0	0.85279	-0.20328

TABLE I. Fitting constants a and b (see text for definition) retrieved for all the curves given in Fig. 2

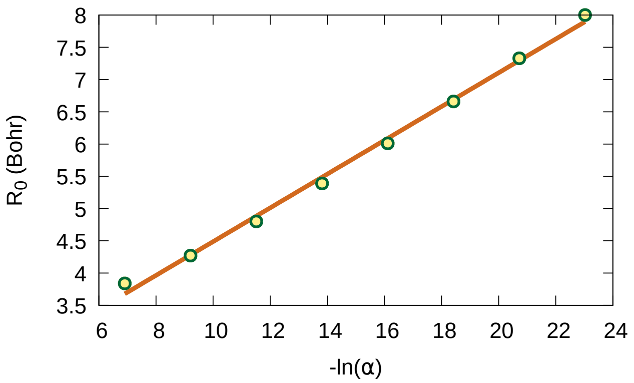

FIG. 3. Spatial position of the step in the Kohn-Sham potential for the Li ion with $2+\alpha$ electrons (see Legend), versus $-\ln(\alpha)$. The potentials were obtained by numerical inversion, relying on local density approximation (LDA) integer-electron densities.

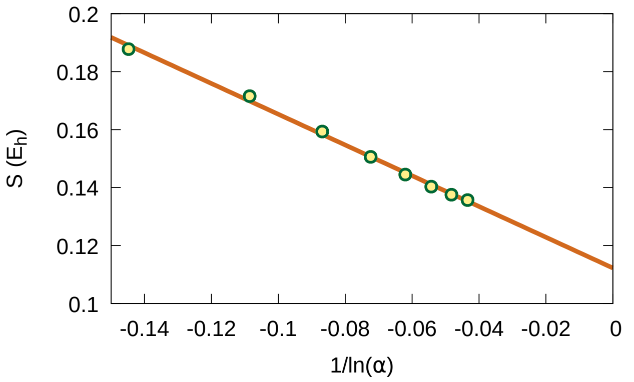

FIG. 4. Height of the step in the Kohn-Sham potential for the Li ion with $2+\alpha$ electrons (see Legend), versus $1/\ln(\alpha)$. The potentials were obtained by numerical inversion, relying on local density approximation (LDA) integer-electron densities.

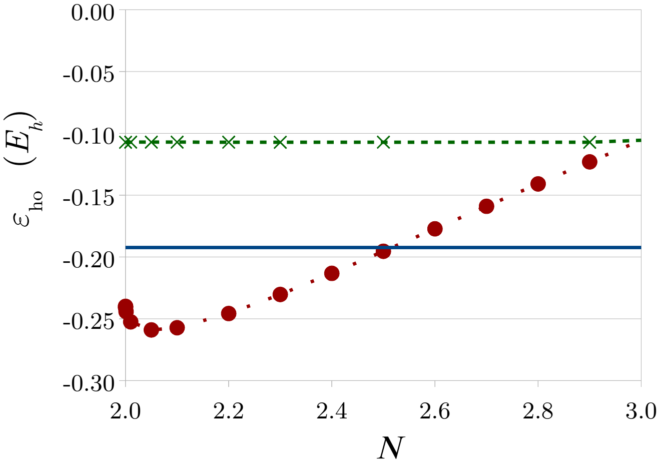

FIG. 5. Highest occupied energy level for the Li ion with $N = 2+\alpha$ electrons, as a function of N, obtained from standard LDA calculations (dark red circles) and for invLDA potentials (green x's). The negative of the ionization potential obtained from LDA total energy differences is given for comparison (solid blue line).

by us to the fact that the piecewise-linearity criterion for the density is enforced by the above inversion procedure.

Alignment of the KS potentials of Fig. 1 by subtracting from each of the potentials the corresponding constant b given in Table I and further subtracting the KS potential for $N = 2$, provides Fig. 11 of the main text. As $\alpha \to 0^+$, the invLDA KS potential forms a plateau around the origin, whose height approaches a constant value and whose width broadens, for the plateau to eventually fill all space and appear as a uniform shift.

The dependence of the plateau height S and width R_0 on α are given in Figs. 3 and 4. The step width is determined manually from the graphs for the KS potentials, observing the position of the dip in the potential, with the accuracy of ~ 0.03 Bohr. From Fig. 3 it is clear that the width of the plateau grows logarithmically, as the number of electrons approaches an integer from above. The convergence of the step height S seems to be proportionate to $1/\ln(\alpha)$.

Back to the alignment of the KS potentials, the second choice for alignment presented in the main text is such that for each value of α the system satisfies the ionization potential (IP) theorem, namely that the highest occupied (ho) KS energy level, $\varepsilon_{\rm ho}(\alpha)$, equals the negative of the LDA IP calculated from total energy differences, for all α. In the case of Li, $I_{\rm LDA} = E({\rm Li}^+) - E({\rm Li}) = -7.142178 - (-7.334610) = 0.192432$ Hartree. Figure 5 shows with green x's the ho energy levels for the invLDA potentials given in Fig. 11 of the main text (and described here as the first alignment choice) along with the ho energy levels obtained from the standard LDA runs for systems with fractional N (dark red circles) and the reference level of (the negative of) the LDA IP (solid blue line). Interestingly, unlike the standard LDA results, which change significantly with α, the invLDA re-

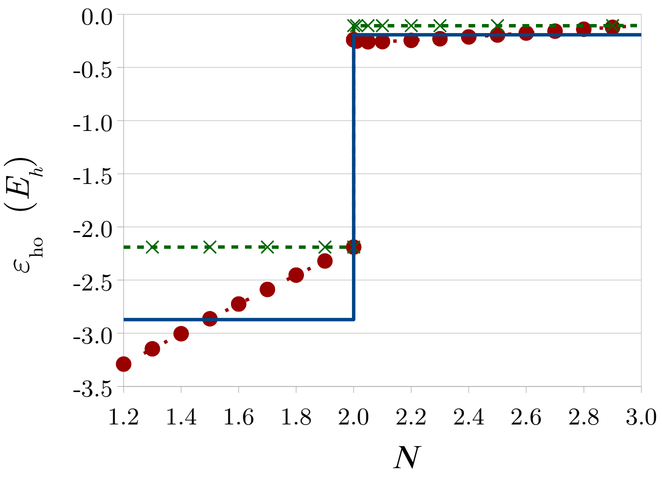

FIG. 6. Same as Fig. 5, for a larger range of N.

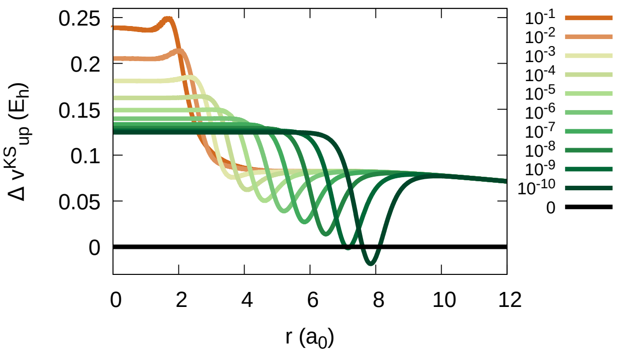

FIG. 7. Difference between the inverted LSDA (invLSDA) KS $\uparrow$-potential for Li with $2+\alpha$ electrons ($1+\alpha$ electrons up and 1 electron down) and the KS potential for Li^+ with 2 electrons, for various values of α (see legend). As $\alpha \to 0^+$, a plateau of height S is formed around the origin.

sults show a *strictly flat* behaviour, as required, although of the wrong height. Therefore, to satisfy the IP theorem, all KS potentials for $N \in (2,3)$ are realigned (after the first alignment procedure detailed above) by just subtracting 0.086832 Hartree. Similarly, for $N \in (1,2)$ the constant 0.681309 Hartree has to be subtracted (see Fig. 6). The resultant graph is given in the main text, Fig. 13.

Performing calculations with the local spin-density approximation (LSDA), namely, associating $1+\alpha$ electrons with the $\uparrow$-channel and 1 electron with the $\downarrow$-channel, we find that $v^{\uparrow}_{\rm KS}(r)$ experiences a plateau, whereas no sharp features are found in $v^{\downarrow}_{\rm KS}(r)$. Aligning the $\uparrow$-potentials such that they all approach zero at infinity, in the same manner detailed above for the LDA, one obtains Fig. 7. The optimal inversion parameters for the LSDA runs were found to be: $p_\uparrow = 0.5$, $\mu_\uparrow = 0.05$, $p_\downarrow = 9.0$, $\mu_\downarrow = 0.03$.

Similar is the situation for the Perdew-Burke-

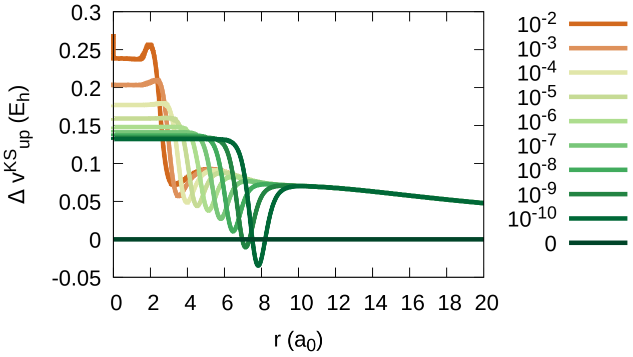

FIG. 8. Difference between the inverted PBE (invPBE) KS $\uparrow$-potential for Li with $2+\alpha$ electrons ($1+\alpha$ electrons up and 1 electron down) and the KS potential for Li^+ with 2 electrons, for various values of α (see legend). As $\alpha \to 0^+$, a plateau of height S is formed around the origin.

Ernzerhof (PBE) Generalized Gradient Approximation (GGA): for the $\uparrow$-channel a plateau is found and shown in Fig. 8; no plateaus are found for the $\downarrow$-channel. The optimal inversion parameters are the same as in LSDA runs.
